# Factors associated with nurses’ physical and psychological quality of life during the COVID-19 pandemic: a multicentre analytical cross-sectional study

**DOI:** 10.1186/s12912-026-04882-8

**Published:** 2026-06-11

**Authors:** Francisco Sampaio, Ricardo Salgado, Philippe Schneider, Jonathan Jubin, Line Martin, Naomi Kabwiku, Philippe Delmas, Ingrid Gilles, Annie Oulevey Bachmann, Claudia Huber, Marie-Chantal Loiselle, Jessica Rassy, Claudia Ortoleva Bucher

**Affiliations:** 1 RISE-Health, Nursing School, University of Porto, Porto, Portugal; 2 La Source School of Nursing, HES-SO University of Applied Sciences and Arts Western Switzerland, Lausanne, Switzerland; 3 Lausanne University Hospital, Lausanne, Switzerland; 4 School of Health Sciences Fribourg, HES-SO University of Applied Sciences and Arts Western Switzerland, Fribourg, Switzerland; 5 School of Nursing, Faculty of Medicine and Health Sciences, University of Sherbrooke, Sherbrooke, Québec Canada

**Keywords:** Quality of life, Health, Nurses, COVID-19, Pandemics, Associated factors, Salutogenesis

## Abstract

**Background:**

COVID-19 has posed unprecedented challenges to healthcare systems worldwide and has adversely affected nurses’ well-being. Although many studies have examined nurses’ mental health and both professional quality of life (ProQOL) and health-related quality of life (HRQoL) during the pandemic, multicentre analyses that simultaneously model physical and psychological quality of life (QoL) domains, with comprehensive adjustment for individual- and organisational-level variables, remain scarce. A more holistic understanding of the factors associated with nurses’ QoL is needed to inform targeted interventions. We aimed to examine associations between perceived stress, resilience, social and organisational support, satisfaction with work quality, and nurses’ physical and psychological QoL during the COVID-19 pandemic.

**Methods:**

This multicentre analytical cross-sectional study was conducted in Switzerland, France, Canada (Québec) and Portugal in autumn 2021. Data were collected using an online self-administered questionnaire administered via Sphinx IQ2 software. Physical and psychological QoL and the independent variables of interest were measured using validated instruments. We used linear regression to examine associations between variables.

**Results:**

Higher perceived stress was consistently associated with lower physical and psychological QoL across all countries. Greater social support and resilience were associated with better psychological QoL in each country. In descriptive analyses, nurses in Switzerland reported the highest QoL scores, whereas those in France reported the lowest.

**Conclusions:**

These findings underline the importance of addressing perceived stress and strengthening psychosocial resources (including social support and resilience) to support nurses’ QoL during public health emergencies. Healthcare institutions should consider stress-management and resilience-building initiatives and foster supportive work environments. Longitudinal studies are needed to clarify temporal relationships and inform context-sensitive interventions.

**Clinical trial number:**

Not applicable.

## Background

The COVID-19 pandemic has arguably been the most significant global crisis since the Second World War, profoundly affecting human society [1, 2]. As a public health emergency, it has had far-reaching consequences for societies and economies, particularly for vulnerable populations worldwide [3]. Healthcare workers, especially those in direct contact with individuals infected by SARS-CoV-2, faced heightened risks of psychological distress during the pandemic, and the global health care workforce was substantially affected [4–7]. A large cross-sectional study conducted during the COVID-19 pandemic suggested that nurses were more likely than physicians to report adverse mental health symptoms, including depression, anxiety and insomnia [8]. A substantial body of research, including systematic reviews and meta-analyses, has therefore examined nurses’ mental health during the pandemic, most commonly focusing on anxiety, depression and post-traumatic stress symptoms [5–7, 9].

However, relatively few studies have examined broader well-being indicators, such as quality of life, from a salutogenic perspective. In this context, a salutogenic perspective refers to an orientation that focuses on factors and resources that support health and well-being, rather than solely on pathology or distress [10]. The World Health Organization defines quality of life as “individuals’ perceptions of their position in life in the context of the culture and value systems in which they live and in relation to their goals, expectations, standards, and concerns” [11]. This multifaceted concept encompasses domains such as physical health and psychological state [11]. While both professional quality of life (ProQOL) and overall/health-related quality of life (HRQoL) have been widely studied among nurses during the COVID-19 pandemic, findings vary across studies, likely reflecting differences in instruments (e.g., ProQOL vs. SF-36/WHOQOL-BREF), settings, time points and data collection approaches [12–19]. Moreover, much of the evidence is cross-sectional and often single-centre, and relatively few investigations have jointly modelled nurses’ physical and psychological quality of life domains with comprehensive adjustment for individual and organisational variables.

Several variables may be associated with nurses’ physical and psychological quality of life, both related and unrelated to the COVID-19 pandemic. Stress is one such variable. For example, a cross-sectional study conducted in December 2020 reported that job stress alone accounted for 27.9% of the variance in nurses’ overall quality of life scores [14]. In addition, high psychological resilience has been associated with improved professional quality of life among nurses [15, 20]. Social support, defined as the help or resources an individual receives from others, or the general willingness of those around them (such as friends and family) to provide psychological and material assistance [21], is another crucial variable that significantly affects all dimensions of professional quality of life [15, 22].

Organisational variables are also relevant. The managerial skills of team leaders were highlighted as an important resource, not only for organising work during the crisis but also for managing conflicts and interpersonal relationships [23]. Communication, work organisation, and support measures implemented by management (e.g., provision of protective equipment, creation of spaces for discussion) or within teams (e.g., team building, emotional support) during and after the crisis also emerged as moderators of the stress perceived by teams in both the short and long term [24–27]. Moreover, trust in hierarchy and management to handle the crisis and ensure staff safety played a key role [26, 28]. Similar to social support, organisational support is positively correlated with compassion, satisfaction and inversely related to burnout, both of which are key dimensions of professional quality of life [29]. Furthermore, higher job satisfaction among nurses is associated with improved quality of life [30]. Exposure to COVID-19 (e.g., being a frontline nurse) is another important variable, as frontline nurses appear to experience more adverse psychological and somatic impacts than their non-frontline counterparts [31]. Finally, professional experience also seems relevant; a 2023 study reported that nurses with 10 or fewer years of experience were more likely to feel emotionally drained, fatigued and burned out [32].

Taken together, the literature indicates that while both ProQOL and HRQoL have been widely investigated in nurses during the pandemic [12–19], studies are methodologically diverse and comparatively few adopt multicentre designs that simultaneously examine physical and psychological QoL domains alongside individual and organisational variables. Given this heterogeneity and the cross-national differences in recruitment context, the present study had an exploratory analytical orientation rather than a strictly confirmatory one.

Accordingly, the aim of this study was to examine the associations between perceived stress, resilience, social support, organisational support, satisfaction with work quality, and nurses’ physical and psychological QoL during the COVID-19 pandemic. Based on previous literature and a salutogenic perspective, we expected perceived stress to be inversely associated with physical and psychological QoL, whereas resilience, social support, organisational support, and satisfaction with work quality would be positively associated with QoL.

## Methods

### Design and population

This multicentre analytical cross-sectional study was conducted in Switzerland, France, Canada (Québec), and Portugal. Data collection occurred as follows: in French-speaking Switzerland (Romandy) from 1 September to 30 November 2021; in German-speaking Switzerland from 1 September to 31 October 2021; in France from 1 September to 30 November 2021; in Portugal from 1 November 2021 to 31 January 2022; and in Canada (Québec) from 1 October to 30 November 2021. At that time, these countries were experiencing their fourth or fifth wave of the COVID-19 pandemic [33]. While the number of cases began to rise during this period (from 9.69 million to 11.15 million), the most significant surge occurred in early winter 2021 [33]. Vaccination coverage during this period for a single dose varied between 57% (Switzerland) and 90% (Portugal) across these countries, with Switzerland showing the lowest coverage (67% by the end of autumn) [33].

This study used a mixed recruitment approach because recruitment strategies varied across countries due to differences in local regulations and access to nurses. Specifically, both convenience sampling and voluntary self-selection sampling were used. Recruitment was conducted through employers and professional organisations. Consequently, the study was not designed to directly compare countries; instead, we examined whether similar patterns of association emerged across contexts using country-stratified analyses. These heterogeneous recruitment approaches may have introduced selection bias; therefore, findings should be interpreted primarily as within-country associations. In Switzerland, France and Canada, nurses were invited by email via their employers or the relevant national nursing bodies. In Portugal, the invitation was posted on the Portuguese Council of Nurses website. In all countries, participants accessed an information sheet via a link on the first page of the questionnaire, which outlined the study purpose, potential risks and benefits, and consent arrangements. Participants were asked to read this information carefully and to proceed only if they agreed. At the end of the questionnaire, participants were asked whether they consented to being contacted for follow-up data collection; those who agreed provided an email address. For subsequent data collection, participants received email invitations and up to three reminders at one-week intervals if they had not yet participated. No formal a priori sample size calculation was performed. Instead, the achieved sample size was pragmatic and reflected the number of eligible nurses who responded during the predefined recruitment period in each country. Given the country-stratified and exploratory nature of the analyses, the aim was to examine patterns of association within each national sample rather than to achieve a single pooled target sample size.

The study was conducted in accordance with local legislation and institutional requirements and received approval from the respective ethics committees. Specifically, the study was reviewed and approved by the following bodies: the Ethics Committee of the Portuguese Society of Mental Health Nursing (03b)/MS/2021) – Portugal; the Cantonal Ethics Committee for Research Involving Human Subjects (2020–02845) – Switzerland; the Research Ethics Committee for the Arts and Humanities, Université de Sherbrooke (2021–3062/Loiselle) – Canada; and the National Council of the Order of Nurses – France. All participants provided electronic informed consent prior to their inclusion in the study; consent was indicated by proceeding to the questionnaire after reading the information sheet.

To be eligible for the study, nurses had to meet the following criteria: active engagement in clinical practice in one of the study countries, a regular work contract, a workload of at least half-time, and fluency in the official language of the respective country. Nurses were excluded if they held exclusively managerial positions, were students, or had teaching roles without clinical practice. Data collection was performed via Sphinx IQ2 software (version 7.2.0.2).

The manuscript adheres to the Strengthening the Reporting of Observational Studies in Epidemiology (STROBE) guidelines for cross-sectional studies [34].

### Measures

We used the physical and psychological domains of the World Health Organization Quality of Life-BREF (WHOQOL-BREF) questionnaire [35]. The independent variables of interest were assessed using: [1] the Perceived Stress Scale (PSS) [36]; [2] the 10-item Connor-Davidson Resilience Scale (CD-RISC) [37]; [3] the Multidimensional Scale of Perceived Social Support (MSPSS) [38]; and [4] the Copenhagen Psychosocial Questionnaire (COPSOQ) [39]. Sociodemographic information was collected using a study-specific questionnaire. Validated and/or translated versions of the instruments demonstrated Cronbach’s alpha values consistently above 0.70.

### Dependent variables

The dependent variables were the physical and psychological domains of the WHOQOL-BREF [35]. The WHOQOL-BREF includes 26 items covering four domains: physical health, psychological health, social relationships, and the environment. In this study, the focus was limited to the physical and psychological domains. These two domains were selected for the present analysis because the study aimed to examine the health-related and psychological dimensions of QoL most directly relevant to perceived stress, resilience, and psychosocial resources during the pandemic. The social relationships and environment domains were not included in the regression analyses because closely related constructs, including perceived social support, support from hierarchy and colleagues, organisational support, and satisfaction with work quality, were already included as independent variables of interest. Including the corresponding WHOQOL-BREF domains in the same analytical framework could therefore have introduced conceptual overlap and complicated interpretation. Accordingly, the present findings should be interpreted as domain-specific analyses of physical and psychological QoL, rather than as a complete WHOQOL-BREF profile. This tool measures self-perceived quality of life, with participants responding on a five-point scale ranging from “not at all” to “completely.” Following established guidelines, raw scores were converted to a 0-100 scale, where higher values indicate better quality of life. This instrument was used in the French, German, or Portuguese versions [40–42].

### Independent variables

The Perceived Stress Scale (PSS), developed by Cohen et al. [36], evaluates the degree to which individuals perceive situations in the previous month as stressful, unpredictable, or uncontrollable. The 10-item scale uses a response ranging from 0 (never) to 4 (very often). This instrument was used in the French, German, or Portuguese versions [43–45].

The Connor-Davidson Resilience Scale (CD-RISC), developed by Campbell-Sills [37], measures resilience, which is defined as the ability to adapt to and recover from adversity. Participants rate their responses on a five-point Likert scale from 0 (not at all true) to 4 (true nearly all the time). This instrument was used in the French, German, or Portuguese versions [46–48].

The Multidimensional Scale of Perceived Social Support (MSPSS) [38] assesses support from three sources: family, friends, and significant others. This 12-item questionnaire uses a seven-point Likert scale ranging from 1 (strongly disagree) to 7 (strongly agree). This instrument was used in the validated French or Portuguese version [49, 50] or the translated German version after following the Wild et al. method [51], which involves forward translation, reconciliation, back-translation, expert review, and pretesting to support semantic and conceptual equivalence across language versions.

The Copenhagen Psychosocial Questionnaire (COPSOQ) measures work-related psychosocial factors, including organisational support (three items) and satisfaction with work quality (two items). Social support is assessed using two items addressing support from supervisors and colleagues. Items are rated on a 5-point Likert scale ranging from 1 (to a very large extent) to 5 (to a minimal extent) [39]. In Portugal, the COPSOQ version used includes a “job satisfaction” subscale rather than the “satisfaction with work quality” subscale used in the other countries; although conceptually related, these subscales are not identical. Because analyses were conducted separately by country, we interpret this construct within each national context rather than as a basis for direct cross-country comparison. This instrument was used in the French, German, and Portuguese versions [52–54].

The sociodemographic and professional questionnaire captured variables such as (i) gender, (ii) age, (iii) marital status, (iv) parental status, and (v) duration of employment in the current role. COVID-19 exposure was categorised as follows: “None” for no direct exposure, “Indirect” for working in settings where COVID-19 patients were present but not the primary focus, and “Direct” for roles dedicated to COVID-19 patient care. This questionnaire was also translated into French, German, and Portuguese. The questionnaire did not collect standardised information on the specific clinical unit or department in which participants worked (e.g., intensive care, emergency, COVID-19 wards, or medical-surgical units). This reflected cross-country differences in the organisation and naming of clinical services, as well as the fact that, during the pandemic, COVID-19 and non-COVID-19 care pathways were sometimes embedded within the same department or service. COVID-19-related exposure was therefore used as the harmonised professional context variable across countries.

### Data analysis

Data analysis proceeded in three steps. First, we summarised sociodemographic variables and standardised instrument scores. Categorical variables were reported as counts and percentages, and continuous variables as means and standard deviations. Second, internal consistency of multi-item measures was assessed using Cronbach’s alpha coefficients (all > 0.8). Participants with > 10% missing data overall were excluded. Remaining missing values (0.3–1.1% depending on country) were imputed using the country-specific mean (continuous variables) or mode (categorical variables). Third, we used linear regression to examine associations between the dependent and independent variables. We assessed multicollinearity and model assumptions using standard diagnostic procedures, including variance inflation factors (VIFs) and residual diagnostics. Model assumptions were assessed through visual residual diagnostics, including inspection of residual distributions and normal Q-Q plots. No substantial deviations from model assumptions were identified. Multicollinearity was assessed using variance inflation factors, and no multicollinearity concerns were observed (all VIFs < 2). We used a two-sided significance level of 0.05. Analyses were conducted using R (version 4.1.2) in RStudio (version 2022.02.0).

## Results

### Participants’ characteristics

A total of 2,063 eligible responses were collected. The characteristics of the participants are summarised in Table 1. Across all countries, most participants were women (83.9%–87.0%), with men comprising a smaller proportion (11.8%–15.8%) and few identifying otherwise. The age distribution was fairly even, although the 40–49 age group was most represented in France (29.7%), and participants aged 50 years or older were more common in Canada (29.6%). The majority of participants were in a couple (69.6%–75.1%), while single participants accounted for 21.8%–26.9%. Parenthood was most prevalent in France (64.8%) and least prevalent in Switzerland (51.4%).

**Table 1:** Participants characteristics stratified by countries

Sociodemographic variables	France (*n* = 1264)	Portugal (*n* = 336)	Switzerland (*n* = 294)	Canada (*n* = 169)
	Frequency (%)	Frequency (%)	Frequency (%)	Frequency (%)
Gender: Man	14.7	15.8	13.9	11.8
Gender: Woman	85.0	83.9	85.0	87.0
Gender: Describes otherwise	0.2	0.0	0.0	0.6
Age: 18–29 y.o.	16.7	12.2	21.8	16.6
Age: 30–39 y.o.	26.3	32.7	28.6	29.0
Age: 40–49 y.o.	29.7	32.4	27.2	24.6
Age: 50 or more y.o.	26.8	22.6	22.1	29.6
Marital situation: Single	21.8	23.2	26.9	26.0
Marital situation: in Couple	75.1	69.6	70.4	71.0
Marital situation: Other	2.7	6.8	2.4	3.0
Children: Yes	64.8	64.0	51.4	59.8
**Professional context variables**	Frequency (%)	Frequency (%)	Frequency (%)	Frequency (%)
Time in position: less than 2 years	22.8	0.6	18.0	22.5
Time in position: 2–5 years	30.7	8.9	25.9	26.0
Time in position: more than 5 years	46.0	90.2	56.1	42.0
Exposure: None	23.7	27.4	10.9	39.6
Exposure: Indirect	51.4	49.7	41.2	37.9
Exposure: Direct	24.4	22.6	47.6	22.5

Some percentages might not add up to 100% because of non-responses

Tenure in current roles varied significantly, with Portugal reporting the highest proportion (90.2%) of participants in their position for over five years. Conversely, shorter tenures (less than two years) were more common in France (22.8%) and Canada (22.5%). Direct exposure to COVID-19 was highest in Switzerland (47.6%), whereas indirect exposure predominated in France (51.4%) and Portugal (49.7%). The largest proportion of participants with no exposure was observed in Canada (39.6%).

### Dependent and independent variables

As shown in Table 2, the physical quality of life scores were highest in Switzerland (M = 70.01, SD = 17.08) and lowest in France (M = 62.12, SD = 19.10). The psychological quality of life scores followed a similar pattern, with Switzerland again reporting the highest scores (M = 66.81, SD = 17.25) and France reporting the lowest scores (M = 57.79, SD = 18.93).

**Table 2:** Dependent and independent variables stratified by countries

Numeric variables	France (*n* = 1264)	Portugal (*n* = 336)	Switzerland (*n* = 294)	Canada (*n* = 169)
	Mean (sd)	Mean (sd)	Mean (sd)	Mean (sd)
Quality of life, physical (SD) 0-100	62.12 (19.10)	62.66 (18.40)	70.01 (17.08)	62.21 (19.38)
Quality of life, psychological (SD) 0-100	57.79 (18.93)	63.65 (18.09)	66.81 (17.25)	61.28 (18.11)
Perceived stress, mean (SD) 0–4	2.99 (0.67)	3.03 (0.63)	2.93 (0.54)	3.07 (0.61)
Social support, mean (SD) 1–7	5.34 (1.26)	5.68 (1.08)	5.82 (1.02)	5.48 (1.19)
Resilience, mean (SD) 0–4	3.54 (0.67)	3.52 (0.69)	3.78 (0.55)	3.64 (0.64)
Support from hierarchy, mean (SD) 1–5	3.66 (1.41)	3.10 (1.33)	4.25 (1.19)	3.81 (1.42)
Support from colleagues, mean (SD) 1–5	4.40 (1.02)	4.17 (1.00)	4.68 (0.78)	4.53 (0.94)
Satisfaction with work quality, mean (SD) 1–5	3.21 (0.90)	2.87 (0.92)	3.51 (0.84)	3.73 (0.89)

Social support and resilience were highest in Switzerland (M = 5.82, SD = 1.02; M = 3.78, SD = 0.55, respectively). Similarly, satisfaction with work quality was lowest in Portugal (M = 2.87, SD = 0.92) and highest in Canada (M = 3.73, SD = 0.89).

Hierarchical support and support from colleagues were most favourable in Switzerland (M = 4.25, SD = 1.19; M = 4.68, SD = 0.78, respectively). Portugal reported the lowest scores for support from hierarchies (M = 3.10, SD = 1.33), whereas support from colleagues was lowest in Portugal as well (M = 4.17, SD = 1.00).

### Regression analysis

#### Quality of life: physical domain

As presented in Table 3, significant variables associated with physical QoL varied across countries and included age, perceived stress, social support, resilience, satisfaction with work quality, and, in Canada, gender. In Canada, being a woman was associated with lower physical QoL (β = -0.48, *p* < 0.05). Older age groups reported lower physical QoL in France and Canada, with the strongest associations observed among participants aged 50 years or older (France: β = -0.34, *p* < 0.001; Canada: β = -0.70, *p* < 0.01).

**Table 3:** Regression analyses on quality of life measured by WHOQOL-BREF total physical score stratified by countries

Sociodemographic variables	France (*n* = 1264)	Portugal (*n* = 336)	Switzerland (*n* = 294)	Canada (*n* = 169)
	β	β	β	β
Gender (vs. Man): Woman	-0.11	-0.17	-0.07	-0.48*
Gender (vs. Man): Describes otherwise	0.80			-1.37
Age (vs. 18–29): 30–39 y.o.	-0.17*	-0.02	-0.13	-0.61**
Age (vs. 18–29): 40–49 y.o.	-0.18*	-0.25	-0.10	-0.56*
Age (vs. 18–29): 50 or more y.o.	-0.34***	-0.25	-0.02	-0.70**
Marital situation (vs. Couple): Single	-0.11*	0.12	-0.09	-0.05
Marital situation (vs. Couple): Other	0.05	-0.12	-0.14	0.27
Children (vs. No): Yes	-0.05	0.21	-0.01	0.21
**Multiple R** ^**2**^	0.02***	0.08***	**0.02**	0.12**
**COVID-19-related variables**				
Years in position (vs. less than 2 years): 2–5	0.00	0.46	-0.09	0.27
Years in position (vs. less than 2 years): more than 5	0.17**	0.36	-0.09	0.43*
Exposure (vs. Direct): Indirect	0.01	-0.14	-0.10	0.30
Exposure (vs. Direct): None	0.00	0.05	-0.31	0.24
**Multiple R** ^**2**^	0.03***	0.11***	**0.02**	0.18**
**Quality of life-related variables**				
Perceived stress	-0.55***	-0.51***	-0.39***	-0.46***
Social support	0.11***	0.14**	0.17**	-0.11
Resilience	0.07**	0.07	0.07	0.22**
Support from hierarchy	0.03	-0.04	0.05	0.09
Support from colleagues	0.02	0.07	0.03	-0.02
Satisfaction with work quality	0.08**	0.12*	0.23***	-0.06
**Multiple R** ^**2**^	0.48***	0.54***	0.41***	0.47***

Table shows the coefficients for the complete model including all blocks. Significant factors are highlighted in bold

*** <0.001; ** <0.01; * <0.05

Perceived stress was consistently associated with lower physical QoL across all countries (β ranging from − 0.39 to -0.55, *p* < 0.001). Social support was positively associated with physical QoL in France (β = 0.11, *p* < 0.001), Portugal (β = 0.14, *p* < 0.01), and Switzerland (β = 0.17, *p* < 0.01). Resilience showed a significant positive association in France (β = 0.07, *p* < 0.01) and Canada (β = 0.22, *p* < 0.01). Satisfaction with work quality was also positively associated with physical QoL in France (β = 0.08, *p* < 0.01), Portugal (β = 0.12, *p* < 0.05), and Switzerland (β = 0.23, *p* < 0.001).

### Quality of life: psychological domain

Table 4 summarizes the regression analyses for the psychological quality of life domain. Perceived stress emerged as a significant inverse variable across all countries (β ranging from − 0.37 to -0.49, *p* < 0.001). Social support (β = 0.13 to 0.26, *p* < 0.05) and resilience (β = 0.19 to 0.28, *p* < 0.01) were positively associated with psychological quality of life in all countries. Satisfaction with work quality was positively associated with psychological QoL in France (β = 0.07, *p* < 0.01), Portugal (β = 0.11, *p* < 0.01), and Switzerland (β = 0.18, *p* < 0.001), whereas support from hierarchy was negatively associated with psychological QoL only in Portugal (β = -0.09, *p* < 0.05).

**Table 4:** Regression analyses on quality of life measured by WHOQOL-BREF total psychological score stratified by countries

Sociodemographic variables	France (*n* = 1264)	Portugal (*n* = 336)	Switzerland (*n* = 294)	Canada (*n* = 169)
	β	β	β	β
Gender (vs. Man): Woman	-0.10	-0.05	-0.15	-0.31
Gender (vs. Man): Describes otherwise	0.84			-1.24
Age (vs. 18–29): 30–39 y.o.	-0.04	-0.03	0.14	-0.04
Age (vs. 18–29): 40–49 y.o.	-0.05	0.04	0.20	0.12
Age (vs. 18–29): 50 or more y.o.	-0.09	0.03	0.36*	0.10
Marital situation (vs. Couple): Single	-0.02	0.10	-0.25*	-0.16
Marital situation (vs. Couple): Other	-0.01	-0.07	-0.11	0.36
Children (vs. No): Yes	0.00	0.02	-0.06	-0.16
**Multiple R** ^**2**^	0.02***	0.07**	0.05***	0.16***
**COVID-19-related variables**				
Years in position (vs. less than 2 years): 2–5	0.03	0.16	-0.23	0.27
Years in position (vs. less than 2 years): more than 5	0.07	0.21	-0.32*	0.25
Exposure (vs. Direct): Indirect	-0.02	-0.01	-0.10	-0.19
Exposure (vs. Direct): None	-0.03	-0.02	-0.26	-0.22
**Multiple R** ^**2**^	0.03***	0.08**	**0.06**	0.19***
**Quality of life-related variables**				
Perceived stress	-0.45***	-0.49***	-0.37***	-0.41***
Social support	0.15***	0.26***	0.22***	0.13*
Resilience	0.28***	0.20***	0.19***	0.20**
Support from hierarchy	0.01	-0.09*	-0.03	0.08
Support from colleagues	0.03	0.07	0.07	0.06
Satisfaction with work quality	0.07**	0.11**	0.18***	0.05
**Multiple R** ^**2**^	0.56***	0.70***	0.51***	0.59***

Table shows the coefficients for the complete model including all blocks. Significant factors are highlighted in bold

*** <0.001; ** <0.01; * <0.05

Gender and age showed inconsistent associations across countries. For example, older age (50 + years) was positively associated with psychological quality of life in Switzerland (β = 0.36, *p* < 0.05), but other countries did not exhibit significant age effects. Marital status and exposure to COVID-19 were generally not significantly associated with psychological quality of life.

## Discussion

In this multicentre cross-sectional study in Switzerland, France, Canada (Québec) and Portugal, we examined associations between perceived stress, resilience, social and organisational support, satisfaction with work quality, COVID-19 exposure, and nurses’ physical and psychological quality of life. The participant profile (predominantly female and mid-career) is consistent with global nursing workforce distributions. In descriptive analyses, QoL scores were highest in Switzerland and lowest in France.

For physical QoL, perceived stress was the only variable consistently associated with lower scores across all countries. This finding is clinically and theoretically plausible. During the COVID-19 pandemic, nurses were exposed to multiple concurrent stressors, including increased workload, rapidly changing protocols, fear of infection, prolonged use of protective equipment, redeployment, moral strain, and repeated exposure to severe illness and death. In a cross-sectional design, these factors cannot be interpreted as causes of lower QoL; nevertheless, they provide a relevant contextual framework for understanding why higher perceived stress may co-occur with poorer physical QoL. Such stressors may be related to fatigue, sleep disturbance, somatic complaints, reduced recovery time, and sustained psychophysiological activation. Perceived stress is particularly relevant because it captures not only exposure to demands, but also the extent to which individuals appraise those demands as unpredictable, uncontrollable, or exceeding available coping resources [36]. The consistency of this association across countries suggests that perceived stress may be an important correlate of reduced physical well-being among nurses during public health emergencies, even when demographic and professional variables are considered [14, 17, 55, 56].

Psychological QoL was also inversely associated with perceived stress in all countries. This association may reflect the cumulative emotional and cognitive burden experienced by nurses during the pandemic, including uncertainty, responsibility for patient safety, disruption of usual care routines, and the emotional demands of caring for patients and families under restrictive infection-control conditions. The absence of a consistent association between COVID-19 exposure and psychological QoL should be interpreted cautiously. COVID-19 exposure was a harmonised variable across countries, but it may not have fully captured the intensity of work-related stressors within specific settings, such as intensive care units, emergency departments, COVID-19 wards, or medical–surgical units. Evidence from the pandemic indicates that clinical setting is relevant to nurses’ psychological well-being because settings differ in workload, patient acuity, mortality exposure, staffing levels, infection risk, and organisational support. Therefore, residual contextual heterogeneity may have remained even after accounting for COVID-19 exposure [56–58].

Resilience was positively associated with psychological QoL across countries. This finding should not be interpreted as suggesting that responsibility for coping lies solely with individual nurses. Rather, resilience is increasingly understood as a dynamic process shaped by individual, relational, and organisational resources. In the present study, higher resilience co-occurred with better psychological QoL, which may be consistent with more adaptive appraisal of stressors, greater perceived manageability, maintenance of professional meaning, and better recovery after adverse events. However, the development and maintenance of resilience also depend on contextual conditions, including supportive leadership, effective communication, collegial connection, access to rest and self-care, and an organisational culture that recognises psychological strain. This interpretation is consistent with evidence showing that resilience among healthcare workers during pandemics is associated with individual coping resources, social and collegial support, professional identity, collaboration, organisational culture, supportive leadership, and opportunities for growth and recovery [58–60].

Social support was positively associated with psychological QoL in all countries, and with physical QoL in most countries. This may reflect the role of relational resources in helping nurses interpret stressful situations as more manageable, regulate emotional responses, and maintain a sense of connectedness during crisis conditions [15, 18, 19, 59–61]. The stronger and more consistent association observed for perceived social support than for support from hierarchy or colleagues may suggest that broader relational resources, including family, friends, and significant others, were particularly relevant to psychological QoL during the pandemic. At the same time, organisational support remains essential, because individual resilience and social support are unlikely to compensate for persistently excessive workload, unsafe staffing, poor communication, or inadequate recovery opportunities. Therefore, interventions should combine individual-level strategies, such as stress management and resilience-building programmes, with organisational measures aimed at reducing avoidable stressors, strengthening team support, ensuring transparent communication, and protecting recovery time.

These results support interventions that address perceived stress and strengthen psychosocial resources (including social support and resilience) among nurses. At the organisational level, fostering supportive work environments and accessible support mechanisms may help promote nurses’ QoL during high-pressure periods, including future public health emergencies.

### Strengths and limitations

This study has several strengths. First, the multicentre design, including Switzerland, France, Canada and Portugal, enhances the generalisability of the findings across diverse healthcare settings. Including nurses from different countries provides a broader understanding of factors associated with physical and psychological QoL during the COVID-19 pandemic. In addition, we used validated and widely recognised instruments (WHOQOL-BREF, PSS, CD-RISC and MSPSS). The high reliability of these measures, as indicated by Cronbach’s alpha values above 0.80, strengthens the robustness of the results.

A further strength is the concurrent assessment of physical and psychological QoL domains using the WHOQOL-BREF across multiple countries, an approach that is less common in multicentre studies, which have often focused on ProQOL or single-setting HRQoL analyses [12–19]. By adopting a salutogenic approach, that is, by focusing on resources associated with better health and well-being rather than solely on adverse symptoms or deficits [10], this study broadens understanding of nurses’ well-being beyond occupational outcomes and highlights the relevance of psychosocial variables such as resilience and social support. Finally, the structured online survey enabled data collection across multiple regions while ensuring participant anonymity, potentially reducing response bias.

Despite these strengths, several limitations should be considered. First, the cross-sectional design precludes causal inference. Second, data were self-reported and may be subject to response bias (including social desirability and recall bias). Third, recruitment strategies differed across countries and may have influenced sample composition, limiting the generalisability of descriptive estimates and introducing selection bias. For example, the Canadian subsample was older and reported lower exposure to COVID-19, which may have affected some associations. Fourth, the online survey format may have excluded nurses with limited digital access, potentially reducing representativeness. Fifth, the exclusive quantitative design may not fully capture the complexity of nurses’ quality of life during crisis situations. Sixth, cultural and system-level differences across countries (including healthcare systems and pandemic responses) may partly account for variation in findings; therefore, direct cross-country comparisons should be made cautiously. We also did not distinguish, in the analyses, between nurses working in public-sector and private-sector organisations, which may have obscured sector-specific differences in working conditions and available support. Similarly, we did not include the clinical unit or department in which nurses worked, because this information was not collected in a sufficiently harmonised way across countries. This may have led to residual confounding, as clinical settings such as intensive care units, emergency departments, COVID-19 wards, and medical-surgical units may involve different levels of workload, patient acuity, mortality exposure, infection risk, moral strain, and organisational support. Future multicentre studies should collect harmonised setting-level variables and, where sample size allows, use stratified or multilevel models to distinguish associations related to COVID-19 exposure from those related to clinical work setting. In addition, no formal a priori sample size calculation was undertaken; therefore, some country-specific analyses, particularly in the smaller Canadian subsample, may have been underpowered to detect weaker associations. We also did not assess potentially important COVID-19-related stressors, such as bereavement following the death of close relatives, which could influence QoL. In Portugal, a different COPSOQ satisfaction subscale was available; consequently, satisfaction should be interpreted within-country. Another limitation is that only the physical and psychological domains of the WHOQOL-BREF were analysed. Although this decision was aligned with the study aim and reduced conceptual overlap with the psychosocial variables included in the regression analyses, it means that the findings do not provide a complete assessment of nurses’ overall QoL. Future studies should consider all four WHOQOL-BREF domains, while ensuring that analytical models avoid conceptual redundancy between variables included in the analyses. Finally, although multilevel modelling can be appropriate for nested data, the study was designed for country-stratified analyses and data were not pooled; future research could apply hierarchical models in harmonised multicountry datasets.

## Conclusions

This study provides insights into factors associated with nurses’ physical and psychological quality of life during the COVID-19 pandemic across four countries. Perceived stress was consistently associated with lower quality of life, whereas resilience and social support were associated with better psychological quality of life. These findings may help inform strategies to strengthen nurses’ well-being and workforce resilience in future public health emergencies and other high-pressure contexts.

Future research should focus on longitudinal designs to clarify temporal relationships and explore the interplay between resilience and other psychosocial factors in different cultural and health care settings. In addition, mixed-methods approaches integrating qualitative components are warranted, as nurses’ narratives are essential to triangulate quantitative findings and to provide a more comprehensive and contextualised understanding of lived experiences during crisis situations. Such studies could inform the development of tailored interventions to increase the well-being of nurses worldwide, particularly during global health crises.

## Data Availability

The data presented in this study are available upon request from the corresponding author. The data are not publicly available due to the presence of health information.

## Abbreviations

CD: RISC–Connor–Davidson Resilience Scale
COPSOQ: Copenhagen Psychosocial Questionnaire
COVID: 19–Coronavirus Disease 2019
HRQoL: Health–related quality of life
MSPSS: Multidimensional Scale of Perceived Social Support
ProQOL: Professional quality of life
PSS: Perceived Stress Scale
QoL: Quality of Life
SARS: CoV–2–Severe Acute Respiratory Syndrome Coronavirus 2
STROBE: Strengthening the Reporting of Observational Studies in Epidemiology
WHO: World Health Organization
WHOQOL: BREF–World Health Organization Quality of Life–BREF
